# Long-term impact of growth hormone therapy on mortality and type 2 diabetes in Prader–Willi syndrome: a nationwide cohort study

**DOI:** 10.3389/fendo.2025.1642129

**Published:** 2025-08-22

**Authors:** Yong Jun Choi, Aram Yang

**Affiliations:** ^1^ Division of Pulmonary and Critical Care Medicine, Department of Internal Medicine, Gangnam Severance Hospital, Yonsei University College of Medicine, Seoul, Republic of Korea; ^2^ Department of Pediatrics, Kangbuk Samsung Hospital, Sungkyunkwan University School of Medicine, Seoul, Republic of Korea

**Keywords:** Prader-Willi syndrome, growth hormone, mortality, diabetes mellitus, type 2, cohort studies

## Abstract

**Background:**

Prader–Willi syndrome (PWS) is a rare genetic disorder characterized by severe multisystem comorbidities and increased mortality. Although growth hormone therapy (GHT) is widely used as standard care, population-based evidence on its long-term safety, particularly in relation to mortality and type 2 diabetes mellitus (T2DM), remains limited. We aimed to investigate the associations between GHT duration, mortality, and T2DM incidence in PWS.

**Methods:**

This is a nationwide cohort study using the Korean National Health Insurance Service database. A total of 385 individuals with PWS were identified between January 2005 and February 2023. GHT duration was the primary exposure. All-cause mortality was analyzed using Cox proportional hazards models, and T2DM risk was evaluated using multivariable logistic regression adjusted for age, comorbidities, and GHT duration.

**Results:**

GHT duration did not directly impact mortality (OR 1.00, 95% CI: 0.99–1.00); however, peripheral vascular disease (aOR 10.66, 95% CI: 1.07–106.56), renal disease (aOR 17.45, 95% CI: 1.17–259.93), adrenal insufficiency (aOR 23.90, 95% CI: 3.19–178.34), and behavioral disorders (aOR 29.51, 95% CI: 2.64–329.95) were significant predictors of all-cause mortality. Longer GHT duration was independently associated with higher T2DM risk (aOR 1.06, 95% CI: 1.02–1.11). Older age, age at PWS diagnosis, and comorbidities (peptic ulcer disease, mild liver disease, and diabetes insipidus) were additional risk factors.

**Conclusions:**

GHT was not a direct predictor of mortality in PWS, which was instead influenced by comorbidities. However, its prolonged use was linked to increased T2DM. These findings support individualized risk assessment and metabolic monitoring in patients with PWS receiving GHT.

## 1 Introduction

Prader–Willi Syndrome (PWS) is a rare genetic disorder (OMIM#176270) with an estimated incidence of 1 in 21,000 live births. It results from the lack of expression of paternally inherited genes on chromosome 15q11–q13 and is characterized by hypothalamic dysfunction, hyperphagia, obesity, intellectual disability, and endocrine abnormalities (1).

PWS is an indication for growth hormone therapy (GHT), as its clinical features, such as impaired growth, abnormal body composition, hypotonia, and low energy expenditure, closely resemble those seen in growth hormone deficiency (GHD) unrelated to PWS. Studies have shown that nearly all individuals with PWS exhibit reduced spontaneous growth hormone (GH) secretion and low insulin-like growth factor-1 (IGF)-1 levels, primarily due to hypothalamic dysfunction and potential defects in neuroendocrine convertase 1 (2, 3). In many countries, including the United States, United Kingdom, and European nations, GHT is initiated in patients with PWS during infancy or early childhood to maximize benefits. In South Korea, GHT for PWS has been covered by the national insurance system starting at age 2, recognizing its critical role in managing this condition (4). These include improvements in growth, body composition, motor and cognitive development, and overall quality of life. The long-term benefits of GHT include reduced fat accumulation, increased lean body mass, and improved metabolic and cardiovascular health.

However, GH exerts counter-regulatory effects on insulin action by stimulating hepatic glucose production and reducing peripheral glucose uptake, thereby raising concerns regarding its long-term metabolic safety. In prepubertal children with PWS, GHT has been associated with increases in insulin resistance, with occasional cases of impaired glucose tolerance reported after approximately two years of treatment (5). Data from KIGS registry have documented rare instances of hyperglycemia and type 2 diabetes mellitus (T2DM) in GH-treated PWS (6). In adults, a longitudinal study of 12 GH-treated patients showed a statistically significant rise in fasting glucose and a modest increase in HbA1c (from 5.6% to 5.8%) (7). A meta-analysis focusing on adult GHT further identified a small increase in fasting glucose and trends toward higher insulin resistance (8). By contrast, an Italian cohort study found that disorders of glucose metabolism in PWS were more strongly associated with age and obesity than with GH exposure (9). A recent systematic review similarly reported that GHT improves body composition without significantly impairing glucose homeostasis (10).

However, GH exerts counter-regulatory effects on insulin action by stimulating hepatic glucose production and reducing peripheral glucose uptake, raising concerns about its long-term metabolic safety. In prepubertal children with PWS, GHT has been associated with increased insulin resistance, with occasional cases of impaired glucose tolerance reported after approximately two years of treatment (5). Data from the KIGS registry have documented rare instances of hyperglycemia and type 2 diabetes mellitus (T2DM) in GH-treated individuals with PWS (6). In adults, a longitudinal study of 12 GH-treated patients showed a statistically significant rise in fasting glucose and a modest increase in HbA1c (from 5.6% to 5.8%) (7). A meta-analysis of GH therapy in adults further reported small increases in fasting glucose and a trend toward greater insulin resistance (8). Conversely, an Italian cohort study suggested that glucose metabolism disorders in PWS are more strongly related to age and obesity than to GH exposure (9). A recent systematic review also concluded that GHT improves body composition without significantly impairing glucose homeostasis (10).

These findings suggest that while GH clearly benefits body composition, its long−term metabolic safety and impact on serious outcomes such as all-cause mortality and T2DM remain limited. In addition, how factors such as timing of diagnosis or duration of therapy influence these outcomes is not well understood.

We hypothesized that prolonged exposure to GHT may be associated with altered risks of mortality and T2DM in individuals with PWS. To address this, we conducted a nationwide population-based study to examine the associations between GHT duration and these long-term outcomes, aiming to inform risk stratification and long-term management in this population.

## 2 Methods

### 2.1 Ethics approval and patient consent

The study protocol was approved by the Institutional Review Board of Kangbuk Samsung Hospital (KBSMC 2023-07-036). The requirement for informed consent was waived due to the use of anonymized data.

### 2.2 Data source

This study utilized data from the Korean National Health Insurance Service (NHIS), which covers 97% of the population and includes diagnostic codes, prescription records, procedures, and demographic information. The NHIS database is linked to the National Death Registry, the National Health Screening Program, and the Rare Incurable Disease Registry (11). Under the Rare Disease Management Act, patients with conditions affecting fewer than 20,000 individuals, including PWS, are eligible for financial support and registered under a special reimbursement code (12).

### 2.3 Study population and design

**Figure 1:**
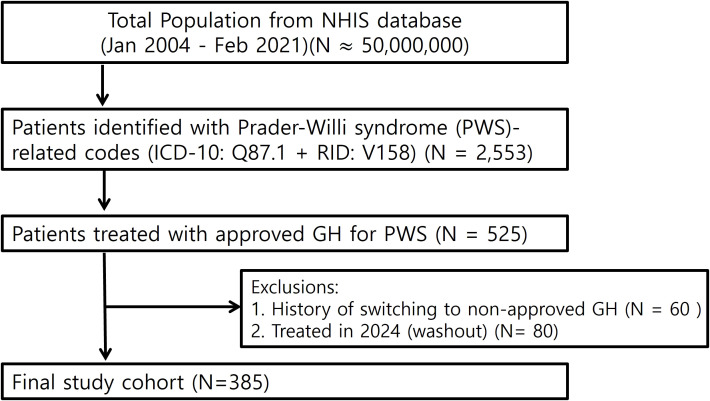
Study flow chart. Flow diagram of patient selection from the NHIS database (January 2004–February 2023). A total of 2,553 patients were identified with Prader–Willi syndrome (PWS)-related codes (ICD-10: Q87.1 and reimbursement code V158). To enhance diagnostic specificity, only patients who received GH products approved for PWS (N = 525) were included. After excluding 60 patients with a history of switching to non-approved GH and 80 patients treated during the 2024 washout period, the final study cohort comprised 385 individuals.

We identified individuals with PWS using the Korean NHIS database from January 2004 to February 2023 using ICD-10 Q87.1 and reimbursement code V158. Because Q87.1 encompasses other congenital syndromes associated with short stature (e.g., Noonan syndrome, Aarskog syndrome), we enhanced diagnostic specificity by including only those treated with somatropin products explicitly approved for PWS: Genotropin (Pfizer), Eutropin (LG Chem), or SciTropin (SciGen Korea). In Korea, such prescriptions require both genetic and clinical confirmation, thus serving as a reliable proxy for diagnosis in NHIS data. Patients treated for other indications (eg, Noonan syndrome), with non-approved GH products, or with GH prescriptions during the designated 2024 washout period were excluded. GH therapy for PWS is typically initiated from age 2 without GH stimulation testing due to its syndromic indication. It is administered at a standard dose of 0.035 mg/kg/day or 1.0 mg/m²/day (maximum 2.7 mg/day), and discontinued when growth velocity falls below 1 cm or near epiphyseal closure. These practices are in line with international guidelines (4) and were applied consistently during the study period. The final study cohort consisted of 385 individuals (**Figure 1**).

### 2.4 Data collection and definitions

Collected variables included sex, age at diagnosis, age at initiation of GHT, duration of GHT, age at last follow-up, and age at death. Comorbidities were identified using International Classification of Diseases, 10th Revision (ICD-10) diagnostic codes, ATC medication codes, and procedure codes, and summarized using the Charlson comorbidity index (CCI) according to Quan’s algorithm (13, 14). Key comorbidities were defined as follows: diabetes insipidus by desmopressin use; behavioral disorders by prescription of psychotropic medications; adrenal insufficiency by ICD-10 codes E27.3 or E27.4; and obstructive sleep apnea (OSA) by diagnosis with concurrent use of continuous positive airway pressure (CPAP). Full definitions and code sets are provided in **Supplementary Table S1**.

### 2.5 Outcome measures

The primary outcome was all-cause mortality, determined through linkage with the National Death Registry. The secondary outcome was the incidence of T2DM, defined by a diagnosis of ICD-10 code E11, followed by the prescription of at least one antidiabetic medication (ATC codes A10A or A10B) within 1 year of diagnosis. The observation period extended from January 2005 to February 2023, capturing a maximum follow-up duration of 16 years.

### 2.6 Statistical analyses

Categorical variables are summarized as frequencies and percentages and were analyzed using chi-square or Fisher’s exact tests. Continuous variables are presented as means ± standard deviations or medians with interquartile ranges (IQRs), depending on their distribution, and were compared using Student’s t-test or the Wilcoxon rank-sum test. Univariate and multivariate logistic regression analyses were conducted to identify risk factors for mortality and T2DM. Variables with a univariable *P <*0.05 were included in the multivariable models. Results are reported as odds ratios (ORs) with 95% confidence intervals (CIs). Statistical significance was defined as a two-sided *P <*0.05. All analyses were conducted using R (version 3.6.1; R Foundation for Statistical Computing, Vienna, Austria).

## 3 Results

### 3.1 Baseline characteristics

During the study period, 385 patients with PWS were included in the analysis. Among them, 207 (53.8%) were male. The median age at diagnosis was 1.0 years (interquartile range [IQR]: 0.0–3.0), and median age at last follow-up was 11.0 years (IQR: 7.0–16.0). **Table 1** presents the baseline characteristics of the patients, categorized based on mortality and the occurrence of T2DM.

**Table 1:** Baseline characteristics of patients with Prader–Willi syndrome by mortality and T2DM.

Group	Total	Survival (*n*=374)	Deceased (*n*=11)	*P*-value	No Type 2 DM (*n*=328)	Type 2 DM (*n*=57)	*P*-value
Sex (male), *n* (%)	207 (53.8%)	200 (53.5%)	7 (63.6%)	0.556	172 (52.4%)	35 (61.4%)	0.267
Age at last follow-up, years	11.0 (7.0;16.0)	11.0 (7.0;15.0)	20.0 (16.5;26.5)	<0.001	10.0 (7.0;14.0)	21.0 (15.0;25.0)	<0.001
Age at diagnosis of PWS, years	1.0 (0.0;3.0)	1.0 (0.0; 3.0)	9.0 (6.0;12.0)	<0.001	0.0 (0.0; 1.0)	7.0 (1.0;10.0)	<0.001
PWS diagnosis age groups, years				<0.001			<0.001
0-2	281 (73.0%)	279 (74.6%)	2 (18.2%)		264 (80.5%)	17 (29.8%)	
2-5	34 (8.8%)	33 (8.8%)	1 (9.1%)		24 (7.3%)	10 (17.5%)	
5-8	27 (7.0%)	25 (6.7%)	2 (18.2%)		18 (5.5%)	9 (15.8%)	
8≥	43 (11.2%)	37 (9.9%)	6 (54.5%)		22 (6.7%)	21 (36.8%)	
Age at start of GHT, years	2.0 (2.0;5.0)	2.0 (2.0; 5.0)	9.0 (6.5;13.5)	<0.001	2.0 (2.0; 4.0)	7.0 (3.0;11.0)	<0.001
Duration of GHT, years	4.7 (2.8;8.4)	4.7 (2.9;8.4)	4.6 (2.2;6.8)	0.525	4.7 (3.0;8.4)	8.0 (5.0;10.7)	0.004
CCI score at PWS diagnosis	0.0 (0.0;1.0)	0.0 (0.0; 1.0)	1.0 (0.0; 1.5)	0.232	0.0 (0.0; 1.0)	1.0 (1.0; 2.0)	<0.001
Peripheral vascular disease	1 (0.3%)	1 (0.3%)	0 (0.0%)	1.000	1 (0.3%)	0 (0.0%)	1.000
Chronic pulmonary disease	127 (33.0%)	122 (32.6%)	5 (45.5%)	0.571	96 (29.3%)	31 (54.4%)	<0.001
Rheumatic disease	1 (0.3%)	1 (0.3%)	0 (0.0%)	1.000	1 (0.3%)	0 (0.0%)	1.000
Peptic ulcer disease	12 (3.1%)	12 (3.2%)	0 (0.0%)	1.000	8 (2.4%)	4 (7.0%)	0.155
Mild liver disease	38 (9.9%)	36 (9.6%)	2 (18.2%)	0.671	25 (7.6%)	13 (22.8%)	0.001
Hemiplegia or paraplegia	29 (7.5%)	29 (7.8%)	0 (0.0%)	0.703	22 (6.7%)	7 (12.3%)	0.230
Renal disease	0 (0.0%)	0 (0.0%)	0 (0.0%)	1.000	0 (0.0%)	0 (0.0%)	1.000
Any malignancy without metastasis	1 (0.3%)	1 (0.3%)	0 (0.0%)	1.000	0 (0.0%)	1 (1.8%)	0.321
Moderate or severe liver disease	2 (0.5%)	2 (0.5%)	0 (0.0%)	1.000	2 (0.6%)	0 (0.0%)	1.000
Metastatic solid tumor	0 (0.0%)	0 (0.0%)	0 (0.0%)	1.000	0 (0.0%)	0 (0.0%)	1.000
T2DM at PWS diagnosis	10 (2.6%)	9 (2.4%)	1 (9.1%)	0.680			

PWS, Prader–Willi syndrome; T2DM, type 2 diabetes mellitus; GHT, growth hormone treatment; AIDS, acquired immune deficiency syndrome.

Comparison based on survival status revealed that the median age at PWS diagnosis was significantly higher in deceased patients than in survivors (9.0 years [IQR: 6.0–12.0] vs. 1.0 years [IQR: 0.0–3.0], *P* < 0.001), and the last follow-up age was also longer in deceased patients (20.0 year [IQR: 16.5–26.5] vs. 11.0 year [IQR: 7.0–15.0], *P* < 0.001). Furthermore, the median age at GH initiation was significantly higher in deceased patients than in survivors (9.0 years [IQR: 6.5–13.5] vs. 2.0 years [IQR: 2.0–5.0], *P* < 0.001), while the duration of GH treatment did not differ significantly between the groups (4.6 years [IQR: 2.2–6.8] vs. 4.7 years [IQR: 2.9–8.4], *P* = 0.525). T2DM prevalence at the time of PWS diagnosis did not differ significantly between survivors and deceased patients (*P* = 0.680).

Patients who developed T2DM were diagnosed with PWS at a significantly older age (7.0 years [IQR: 1.0–10.0] vs. 0.0 years [IQR: 0.0–1.0], P < 0.001), and had a higher age at last follow-up (21.0 years [IQR: 15.0–25.0] vs. 10.0 years [IQR: 7.0–14.0], P < 0.001) compared to those without T2DM. Additionally, the T2DM group had a higher age at the start of GHT and a longer duration of GHT compared to the non-T2DM group (7.0 years [IQR: 3.0–11.0] vs. 2.0 years [IQR: 2.0–4.0], *P* < 0.001, and 8.0 years [IQR: 5.0–10.7] vs. 4.7 years [IQR: 3.0–8.4], *P* = 0.004, respectively). In terms of underlying diseases at PWS diagnosis, the T2DM group had a higher CCI score (1.0 [IQR: 1.0–2.0] vs. 0.0 [IQR: 0.0–1.0], *P* < 0.001) and a higher prevalence of chronic pulmonary disease (54.4% [n=31] vs. 29.3% [n=96], *P* < 0.001) and mild liver disease (22.8% [n=13] vs. 7.6% [n=25], *P* = 0.001) compared to the non-T2DM group.

### 3.2 Comorbidities and clinical outcomes

**Table 2** presents the clinical outcomes and the occurrence of comorbidities at the end of the observation period. Non-survivors had significantly higher CCI scores (median: 3.0 [IQR: 2.0–6.0]) than survivors (median: 2.0 [IQR: 1.0–3.0], *P* = 0.029). Additionally, non-survivors had significantly higher occurrences of T2DM (45.5% [n=5] vs. 13.9% [n=52], *P* = 0.013), peripheral vascular disease (18.2% [n=2] vs. 1.9% [n=7], *P* = 0.012), renal disease (18.2% [n=2] vs. 1.1% [n=4], *P* = 0.001), behavior disorders (90.9% [n=10] vs. 24.6% [n=92], *P* < 0.001), OSA (36.4% [n=4] vs. 10.7% [n=40], *P* = 0.031), and adrenal insufficiency (18.2% [n=2] vs. 0.8% [n=3], *P* < 0.001) compared to survivors.

**Table 2:** Distribution of comorbidities in patients with Prader–Willi syndrome by survival and T2DM status.

Group	Total	Survival (*n*=374)	Deceased (*n*=11)	*P*-value	No Type 2 DM (*n*=328)	Type 2 DM (*n*=57)	*P*-value
Death at last follow-up	NA	0 (0.0%)	11 (100.0%)	NA	6 (1.8%)	5 (8.8%)	0.013
T2DM at last follow-up	57 (14.8%)	52 (13.9%)	5 (45.5%)	0.013	0 (0.0%)	57 (100.0%)	NA
Age at death, years			20.0 (16.5;26.5)		19.5 (17.0; 21.0)	28.0 (16.0; 28.0)	0.582
Death at last follow-up	NA	0 (0.0%)	11 (100.0%)	NA	6 (1.8%)	5 (8.8%)	0.013
Age at T2DM diagnosis, years	14.3 ± 3.8	14.4 ± 3.9	13.2 ± 2.8	0.506	–	14.3 ± 3.8	
CCI score at last follow-up	2.0 (1.0;3.0)	2.0 (1.0; 3.0)	3.0 (2.0; 6.0)	0.029	2.0 (1.0; 3.0)	4.0 (3.0; 6.0)	<0.001
Peripheral vascular disease	9 (2.3%)	7 (1.9%)	2 (18.2%)	0.012	5 (1.5%)	4 (7.0%)	0.040
Chronic pulmonary disease	357 (92.7%)	346 (92.5%)	11 (100.0%)	0.724	301 (91.8%)	56 (98.2%)	0.144
Rheumatic disease	4 (1.0%)	4 (1.1%)	0 (0.0%)	1.000	2 (0.6%)	2 (3.5%)	0.199
Peptic ulcer disease	78 (20.3%)	75 (20.1%)	3 (27.3%)	0.836	45 (13.7%)	33 (57.9%)	<0.001
Mild liver disease	106 (27.5%)	100 (26.7%)	6 (54.5%)	0.091	68 (20.7%)	38 (66.7%)	<0.001
Hemiplegia or paraplegia	104 (27.0%)	104 (27.8%)	0 (0.0%)	0.089	93 (28.4%)	11 (19.3%)	0.208
Renal disease	6 (1.6%)	4 (1.1%)	2 (18.2%)	0.001	3 (0.9%)	3 (5.3%)	0.062
Any malignancy without metastasis	1 (0.3%)	1 (0.3%)	0 (0.0%)	1.000	0 (0.0%)	1 (1.8%)	0.321
Moderate or severe liver disease	2 (0.5%)	2 (0.5%)	0 (0.0%)	1.000	2 (0.5%)	0 (0.0%)	1.000
Metastatic solid tumor	1 (0.3%)	1 (0.3%)	0 (0.0%)	1.000	1 (0.3%)	0 (0.0%)	1.000
Hypothyroidism, *n* (%)	51 (13.2%)	49 (13.1%)	2 (18.2%)	0.969	45 (13.7%)	6 (10.5%)	0.657
AED medication, *n* (%)	48 (12.5%)	46 (12.3%)	2 (18.2%)	0.905	26 (7.9%)	22 (38.6%)	<0.001
Behavior disorder, *n* (%)	102 (26.5%)	92 (24.6%)	10 (90.9%)	<0.001	67 (20.4%)	35 (61.4%)	<0.001
Hormone replacement therapy, *n* (%)	19 (4.9%)	18 (4.8%)	1 (9.1%)	1.000	13 (4.0%)	6 (10.5%)	0.075
Diabetes insipidus, *n* (%)	17 (4.4%)	15 (4.0%)	2 (18.2%)	0.131	9 (2.7%)	8 (14.0%)	0.001
Bariatric surgery, *n* (%)	1 (0.3%)	1 (0.3%)	0 (0.0%)	1.000	0 (0.0%)	1 (1.8%)	0.321
Obstructive sleep apnea, *n* (%)	44 (11.4%)	40 (10.7%)	4 (36.4%)	0.031	31 (9.5%)	13 (22.8%)	0.007
Adenotonsillectomy, *n* (%)	34 (8.8%)	33 (8.8%)	1 (9.1%)	1.000	28 (8.5%)	6 (10.5%)	0.814
Adrenal insufficiency, *n* (%)	5 (1.3%)	3 (0.8%)	2 (18.2%)	<0.001	2 (0.6%)	3 (5.3%)	0.026

PWS, Prader–Willi syndrome; T2DM, type 2 diabetes mellitus; GHT, growth hormone treatment; AIDS, acquired immune deficiency syndrome; AED, antiepileptic drug; CPAP, continuous positive airway pressure.

In the comparison between T2DM and non-T2DM patients, there was no significant difference in age at death (19.5 vs. 28.0 years, *P* = 0.582). However, those with T2DM had significantly higher CCI scores (4.0 [IQR: 3.0–6.0] vs. 2.0 [IQR: 1.0–3.0], *P* < 0.001) and a higher prevalence of peripheral vascular disease (7.0% [n=4] vs. 1.5% [n=5], *P* = 0.040), peptic ulcer disease (57.9% [n=33] vs. 13.7% [n=45], *P* < 0.001), and mild liver disease (66.7% [n=38] vs. 20.7% [n=68], *P* < 0.001). Furthermore, patients with T2DM had higher AED medication use (38.6% [n=22] vs. 7.9% [n=26], *P* < 0.001), behavioral disorders (61.4% [n=35] vs. 20.4% [n=67], *P* < 0.001), diabetes insipidus (14.0% [n=8] vs. 2.7% [n=9], *P* = 0.001), OSA (22.8% [n=13] vs. 9.5% [n=31], *P* = 0.007), and adrenal insufficiency (5.3% [n=3] vs. 0.6% [n=2], *P* = 0.026).

### 3.3 Risk factors for mortality

**Table 3** presents the results of univariate and multivariate logistic regression analyses for all-cause mortality. In the univariate analysis, older age (OR: 1.024 [95% CI: 1.016–1.032]), later PWS diagnosis (OR: 1.040 [95% CI: 1.024–1.056]), delayed initiation of GHT (OR: 1.048 [95% CI: 1.029–1.068]), and higher CCI scores (OR: 1.072 [95% CI: 1.036–1.110]) were associated with all-cause mortality. In terms of underlying diseases, peripheral vascular disease (OR: 11.651 [95% CI: 2.118–64.091]), renal disease (OR: 20.556 [95% CI: 3.325–127.072]), behavior disorders (OR: 30.652 [95% CI: 3.872–242.683]), diabetes insipidus (OR: 5.319 [95% CI: 1.056–26.792]), T2DM (OR: 5.160 [95% CI: 1.520–17.521]), OSA (OR: 4.771 [95% CI: 1.338–17.016]), and adrenal insufficiency (OR: 27.481 [95% CI: 4.080–185.114]) were significantly associated with all-cause mortality.

**Table 3:** Risk factors associated with mortality in patients with Prader–Willi syndrome.

Variables	Unadjusted	*P*-value	Adjusted	*P*-value
Sex	1.522 (0.438-5.288)	0.508		
Age at last-follow up	1.024 (1.016-1.032)	<0.001	1.023 (0.924-1.132)	0.655
Age at diagnosis of PWS	1.040 (1.024-1.056)	<0.001	1.035 (0.967-1.108)	0.319
Age at start of GHT	1.048 (1.029-1.068)	<0.001	1.036 (0.022-48.492)	0.986
Duration of GHT	0.999 (0.994-1.004)	0.622		
CCI score at last follow-up	1.072 (1.036-1.110)	<0.001		
Peripheral vascular disease	11.651 (2.118-64.091)	0.005	10.656 (1.066-106.559)	0.044
Chronic pulmonary disease	not applicable*	1.000		
Rheumatic disease	not applicable*	1.000		
Peptic ulcer disease	1.495 (0.387-5.772)	0.560		
Mild liver disease	3.288 (0.982-11.012)	0.054		
Hemiplegia or paraplegia	not applicable*	1.000		
Renal disease	20.556 (3.325-127.072)	0.001	17.450 (1.171-259.927)	0.038
Any malignancy without metastasis	not applicable*			
Moderate or severe liver disease	not applicable*			
Metastatic solid tumor	not applicable*			
Hypothyroidism	1.474 (0.309-7.024)	0.626		
AED medication	1.585 (0.332-7.563)	0.564		
Behavior disorder	30.652 (3.872-242.683)	0.001	29.508 (2.639-329.953)	0.006
Hormone replacement therapy	1.978 (0.240-16.304)	0.526		
Diabetes insipidus	5.319 (1.056-26.792)	0.043	5.145 (0.628-42.167)	0.128
T2DM	5.160 (1.520-17.521)	0.009	4.727 (0.047-475.529)	0.514
Obstructive sleep apnea	4.771 (1.338-17.016)	0.016	4.544 (0.127-161.930)	0.407
Adrenal insufficiency	27.481 (4.080-185.114)	<0.001	23.897 (3.186-178.340)	0.002

PWS, Prader–Willi syndrome; T2DM, type 2 diabetes mellitus; GHT, growth hormone treatment; CCI, Charlson comorbidity index; AED, antiepileptic drug.

The odds ratio (OR) and 95% confidence interval (CI) could not be estimated because all events occurred in one group or none in either group.

In the adjusted analysis, peripheral vascular disease (OR: 10.656 [95% CI: 1.066–106.559]), renal disease (OR: 17.450 [95% CI: 1.171–259.927]), behavior disorders (OR: 29.508 [95% CI: 2.639–329.953]), and adrenal insufficiency (OR: 23.897 [95% CI: 3.186–178.340]) showed significant associations with all-cause mortality. However, age and GH use were not significantly associated with all-cause mortality (**Table 3**).

### 3.4 Risk factors for T2DM

**Table 4** presents the results of the logistic regression analysis for factors associated with the occurrence of T2DM. In the unadjusted analysis, older age (OR: 1.120 [95% CI: 1.099–1.141]), later PWS diagnosis (OR: 1.148 [95% CI: 1.111–1.186]), older age at GHT initiation (OR: 1.149 [95% CI: 1.107–1.192]), longer GHT duration (OR: 1.065 [95% CI: 1.026–1.105]), and higher CCI score (OR: 1.485 [95% CI: 1.177–1.874]) were significantly associated with T2DM occurrence. Among underlying diseases, several factors were significantly associated with T2DM occurrence, including peripheral vascular disease (OR: 4.875 [95% CI: 1.268–18.741]), peptic ulcer disease (OR: 8.647 [95% CI: 4.686–15.958]), mild liver disease (OR: 7.647 [95% CI: 4.147–14.102]), and renal disease (OR: 6.019 [95% CI: 1.184–30.596]). Additionally, the use of AEDs (OR: 7.301 [95% CI: 3.747–14.225]), behavior disorders (OR: 6.197 [95% CI: 3.411–11.259]), HRT use (OR: 2.851 [95% CI: 1.037–7.839]), diabetes insipidus (OR: 5.787 [95% CI: 2.132–15.710]), OSA (OR: 2.831 [95% CI: 1.377–5.820]), and adrenal insufficiency (OR: 9.056 [95% CI: 1.479–55.456]) were also significantly associated with T2DM occurrence.

**Table 4:** Risk factors associated with T2DM in patients with Prader–Willi syndrome.

Variables	Unadjusted	*P*-value	Adjusted	*P*-value
Sex	1.443 (0.811-2.566)	0.212		
Age at last-follow up	1.120 (1.099-1.141)	<0.001	1.107 (1.062-1.154)	<0.001
Age at diagnosis of PWS	1.148 (1.111-1.186)	<0.001	1.128 (1.012-1.258)	0.030
Age at start of GHT	1.149 (1.107-1.192)	<0.001	1.114 (0.701-1.771)	0.648
Duration of GHT	1.065 (1.026-1.105)	0.001	1.063 (1.022-1.105)	0.002
CCI score	1.485 (1.177-1.874)	<0.001		
Peripheral vascular disease	4.875 (1.268-18.741)	0.021	4.044 (0.383-42.663)	0.244
Chronic pulmonary disease	5.023 (0.669-37.724)	0.117		
Rheumatic disease	5.927 (0.818-42.959)	0.078		
Peptic ulcer disease	8.647 (4.686-15.958)	<0.001	7.275 (2.871-18.433)	<0.001
Mild liver disease	7.647 (4.147-14.102)	<0.001	6.543 (2.421-17.683)	<0.001
Hemiplegia or paraplegia	0.604 (0.300-1.217)	0.159		
Renal disease	6.019 (1.184-30.596)	0.031	4.293 (0.000-999.000)	0.892
Any malignancy without metastasis	not applicable*			
Moderate or severe liver disease	not applicable*			
Metastatic solid tumor	not applicable*			
Hypothyroidism	0.740 (0.300-1.824)	0.513		
AED medication	7.301 (3.747-14.225)	<0.001	5.544 (0.443-69.373)	0.184
Behavior disorder	6.197 (3.411-11.259)	<0.001	5.052 (0.455-56.042)	0.175
Hormone replacement therapy	2.851 (1.037-7.839)	0.042	0.875 (0.225-3.397)	0.847
Diabetes insipidus	5.787 (2.132-15.710)	0.001	4.870 (1.186-19.990)	0.028
Obstructive sleep apnea	2.831 (1.377-5.820)	0.005	2.517 (0.408-15.524)	0.320
Adrenal insufficiency	9.056 (1.479-55.456)	0.017	6.942 (0.383-125.876)	0.190

PWS, Prader–Willi syndrome; T2DM, type 2 diabetes mellitus; GHT, growth hormone treatment; CCI, Charlson Comorbidity Index; AED, antiepileptic drug.

The odds ratio (OR) and 95% confidence interval (CI) could not be estimated because all events occurred in one group or none in either group.

However, in the multivariate analysis, older age (OR: 1.107 [95% CI: 1.062–1.154]), later PWS diagnosis (OR: 1.128 [95% CI: 1.012–1.258]), longer GHT duration (OR: 1.063 [95% CI: 1.022–1.105]), peptic ulcer disease (OR: 7.275 [95% CI: 2.871–18.433]), mild liver disease (OR: 6.543 [95% CI: 2.421–17.683]), and diabetes insipidus (OR: 4.870 [95% CI: 1.186–19.990]) showed significant associations with T2DM occurrence.

## 4 Discussion

This study investigated long-term outcomes of GHT on mortality and T2DM in patients with PWS using a nationwide cohort. Our analysis suggests that mortality in this population is more closely associated with comorbidities—especially peripheral vascular disease, renal disease, behavioral disorders, and adrenal insufficiency—than with GHT itself.

The impact of GHT on mortality in PWS remains unclear. Some studies have suggested that early GHT may improve survival (15), others report no significant effect (16–18). Research in patients with idiopathic short stature (ISS), isolated GHD, and small-for-gestational-age (SGA) highlights that GHT may increase long-term mortality related to cardiovascular and hematologic diseases, necessitating ongoing surveillance (19). Studies on PWS have reported adverse events and deaths during GHT, though no direct causal relationship has been established (6, 10). Our findings align with the prevailing consensus that GHT itself does not directly influence mortality rates (20). Instead, early diagnosis of PWS and effective management of comorbidities are crucial in improving survival outcomes.

Among the comorbidities observed, adrenal insufficiency, a common but often underrecognized complication in PWS, may have contributed to stress-related mortality from acute adrenal crisis in some cases (21–23). Although we could not distinguish central from primary adrenal insufficiency using claims data, the observed association with mortality emphasizes the importance of early detection and appropriate hydrocortisone coverage during acute illness or surgery (24). Behavioral disorders were also more common among non-survivors. While not traditionally considered direct mortality drivers, these conditions may increase the risk of choking, gastric perforation, and obesity-related morbidity (25, 26). Consistent with previous studies, respiratory failure remains the leading cause of mortality in PWS (31%–70%) (15, 26). Although no direct link has been confirmed, GHT may exacerbate respiratory dysfunction due to hypothalamic impairment (25), underscoring the need for respiratory assessment in those receiving GHT.

In contrast to mortality, GHT duration was independently associated with a higher risk of T2DM, even after adjusting for age, timing of diagnosis, and comorbid conditions. While GHT improves body composition and reduces visceral adiposity, it may also reduce insulin sensitivity through counter-regulatory mechanisms (27).

Clinical evidence regarding the metabolic effects of GHT has been mixed. Some observational studies have reported no significant deterioration in glucose metabolism during GHT, including in GH-treated children followed into adulthood (28), adults receiving long-term biosimilar GHT (29), and children born SGA (30).

However, other studies in SGA children (31) and adults with GHD (27, 32) have demonstrated impaired insulin action following GH administration. GH-induced lipolysis increases circulating free fatty acids, which interfere with skeletal muscle glucose uptake via the glucose–fatty acid cycle (27), and may promote intramyocellular lipid accumulation that disrupts insulin signaling (33).

In our PWS cohort, older age was independently associated with T2DM, independent of other metabolic factors. Moreover, certain comorbidities, including peptic ulcer disease, mild liver disease, and diabetes insipidus, were associated with an increased risk of T2DM, though their clinical significance in the context of PWS requires further investigation.

A later PWS diagnosis was independently linked to T2DM risk, likely due to prolonged exposure to metabolic risk factors such as obesity and insulin resistance. Early recognition and intervention, including lifestyle modifications and timely GHT initiation, may help reduce long-term metabolic complications.

In other conditions, such as Turner syndrome, GH-related insulin resistance may normalize after discontinuation (34), though large retrospective studies in pediatric populations have reported persistent T2DM (35, 36) and cardiovascular risks post-treatment (37).

The association between GHT and T2DM in PWS remains uncertain due to mixed findings. Several studies have reported low diabetes incidence during GHT (9, 10), including one involving 235 patients with only a single case attributed to preexisting obesity (38). In contrast, other studies have identified abnormal glucose metabolism and insulin resistance, particularly in prepubertal children or those with obesity (5, 6), and slightly impaired glucose homeostasis in adults receiving long-term GHT (8, 39). Thus, it has been established by expert consensus to include a diabetes risk assessment in patients with PWS receiving GHT, especially those who are obese, older than 12 years, or on antipsychotic medications (2, 5, 40).

This study has several strengths, including its large, nationally representative sample and linkage with mortality records. Use of validated diagnostic and treatment codes allowed for reliable cohort construction and longitudinal follow-up. However, several limitations merit consideration. Residual confounding due to unmeasured variables such as body mass index, laboratory data, or family history could not be fully addressed. In addition, the left-skewed distribution of age at PWS diagnosis, particularly among patients without T2DM, may have influenced group comparisons. Although age at diagnosis was included as a covariate in multivariate models, the potential for bias remains. Furthermore, the inability to differentiate PWS genetic subtypes may have limited our ability to explore phenotype–treatment interactions. Lastly, as with all observational studies, the associations observed cannot establish causality. Prospective studies are needed to validate our findings, explore alternative explanations, and inform risk stratification in the management of PWS.

## 5 Conclusion

This nationwide study found that while GHT was not directly associated with increased mortality in patients with PWS, it was linked to a higher likelihood of T2DM with longer treatment duration. These findings highlight the need for long-term, multidisciplinary monitoring of both endocrine and non-endocrine complications in this population. A multidisciplinary approach—including endocrinology, nutrition, behavioral management, and psychosocial support—remains essential to optimizing outcomes in patients receiving GHT.

## Data Availability

The raw data supporting the conclusions of this article will be made available by the authors, without undue reservation.
