# Supplementary material for: Long-term impact of growth hormone therapy on mortality and type 2 diabetes in Prader–Willi syndrome: a nationwide cohort study
Source: Front Endocrinol (Lausanne). 2025 Aug 22;16:1642129. doi: 10.3389/fendo.2025.1642129 (PMC12412060; doi:10.3389/fendo.2025.1642129)
Supplement: Supplementary file 1 [file Table1.docx]

# Supplementary Table 1. Definition of Comorbidities in the Study

| Comorbidity | Definition | Codes |
| --- | --- | --- |
| Myocardial infarction | Charlson Comorbidity Index | ICD-10: I21, I22, I25.2 |
| Congestive heart failure | Charlson Comorbidity Index | ICD-10: I09.9, I11.0, I13.0, I13.2, I25.5, I42.0, I42.5, I42.6, I42.7, I42.8, I42.9, P29.0, I43, I50 |
| Peripheral vascular disease | Charlson Comorbidity Index | ICD-10: I70, I71, I73.1, I73.8, I73.9, I77.1, I79.0, I79.2, K55.1, K55.8, K55.9, Z95.8, Z95.9 |
| Cerebrovascular disease | Charlson Comorbidity Index | ICD-10: G45, G46, I60, I61, I62, I63, I64, I65, I66, I67, I68, I69 |
| Dementia | Charlson Comorbidity Index | ICD-10: F00, F01, F02, F03, G30, F05.1, G31.1 |
| Chronic pulmonary disease | Charlson Comorbidity Index | ICD-10: J40, J41, J42, J43, J44, J45, J46, J47, J60, J61, J62, J63, J64, J65, J66, J67, I27.8, I27.9, J68.4, J70.1, J70.3 |
| Rheumatic disease | Charlson Comorbidity Index | ICD-10: M05, M06, M32, M33, M34, M31.5, M35.1, M35.3, M36.0 |
| Peptic ulcer disease | Charlson Comorbidity Index | ICD-10: K25, K26, K27, K28 |
| Mild liver disease | Charlson Comorbidity Index | ICD-10: B18, K74, K70.0, K70.1, K70.2, K70.3, K70.9, K71.3, K71.4, K71.5, K71.7, K76.0, K76.2, K76.3, K76.4, K76.8, K76.9, Z94.4 |
| Diabetes without chronic complication | Charlson Comorbidity Index | ICD-10: E10.0, E10.1, E10.6, E10.8, E10.9, E11.0, E11.1, E11.6, E11.8, E11.9, E12.0, E12.1, E12.6, E12.8, E12.9, E13.0, E13.1, E13.6, E13.8, E13.9, E14.0, E14.1, E14.6, E14.8, E14.9 |
| Diabetes with chronic complication | Charlson Comorbidity Index | ICD-10: E10.2, E10.3, E10.4, E10.5, E10.7, E11.2, E11.3, E11.4, E11.5, E11.7, E12.2, E12.3, E12.4, E12.5, E12.7, E13.2, E13.3, E13.4, E13.5, E13.7, E14.2, E14.3, E14.4, E14.5, E14.7 |
| Hemiplegia or paraplegia | Charlson Comorbidity Index | ICD-10: G81, G82, G04.1, G11.4, G80.1, G80.2, G83.0, G83.1, G83.2, G83.3, G83.4, G83.9 |
| Renal disease | Charlson Comorbidity Index | ICD-10: N18, N19, I12.0, I13.1, N03.2, N03.3, N03.4, N03.5, N03.6, N03.7, N05.2, N05.3, N05.4, N05.5, N05.6, N05.7, N25.0, Z49.0, Z49.1, Z49.2, Z94.0, Z99.2 |
| Any malignancy, including lymphoma and leukaemia, except malignant neoplasm of skin | Charlson Comorbidity Index | ICD-10: C00, C01, C02, C03, C04, C05, C06, C07, C08, C09, C10, C11, C12, C13, C14, C15, C16, C17, C18, C19, C20, C21, C22, C23, C24, C25, C26, C30, C31, C32, C33, C34, C37, C38, C39, C40, C41, C43, C45, C46, C47, C48, C49, C50, C51, C52, C53, C54, C55, C56, C57, C58, C60, C61, C62, C63, C64, C65, C66, C67, C68, C69, C70, C71, C72, C73, C74, C75, C76, C81, C82, C83, C84, C85, C88, C90, C91, C92, C93, C94, C95, C96, C97 |
| Moderate or severe liver disease | Charlson Comorbidity Index | ICD-10: I85.0, I85.9, I86.4, I98.2, K70.4, K71.1, K72.1, K72.9, K76.5, K76.6, K76.7 |
| Metastatic solid tumour | Charlson Comorbidity Index | ICD-10: C77, C78, C79, C80 |
| AIDS/HIV | Charlson Comorbidity Index | ICD-10: B20, B21, B22, B24 |
| Hypothyroidism | ICD-10 code E02, E03, or E06.3 with thyroid hormone prescription (levothyroxine, liothyronine) | ICD-10: E02, E03, E06.3;  ATC code: 183601ATB, 83602ATB, 183603ATB, 83604ATB, 183606ATB,183607ATB,183608ATB, 183609ATB, 183610ATB, 183611ATB |
| Anti-epileptic drug use (AED) | Use of anti-epileptic drugs for ≥2 weeks | ATC code:  123102ATB, 123102ATR, 123104ATR, 123130ASY, 136401ATB, 181001ATB, 181002ATB, 181003ATB, 181004ATB, 181005ATB, 206301ATB, 206302ATB, 206303ATB, 206330ASS, 241801ACR, 241801ATB, 241802ACR, 241803ACH, 241803ACR, 241803ATB, 241804ACH, 241804ACR, 241804ATB, 247002ACS, 247701ATB, 250601ATB, 611702ATB, 611703ATB, 611704ATB, 611705ATB |
| Cardiac comorbidities | Use of cardiovascular medications | ATC code: 107601ATB, 107602ATB, 107603ATB, 145706ATB, 145707ACR, 145707ATR, 157501ATR, 157503ATR, 180301ATB, 180302ATB, 180303ATB, 182001ATB, 201405ATR, 201409ATR, 201901ATB, 247603ATR, 247605ATR, 247606ATB, 247607ATB, 459801ACH, 459801ATB, 459802ACH, 459901ATB, 459902ATB, 470801ATB, 470802ATB, 483201ATB, 483202ATB, 111402ATB, 111403ATB, 117901ATB, 117902ATB, 117903ATB, 117904ATB, 125001ATB, 125002ATB, 125003ATB, 125004ACR, 125005ATB, 125006ACR, 125007ACR, 125007ATR, 125008ACR, 125008ATR, 144801ATB, 144831AEL, 107401ATB, 159302ATB, 219501ATB, 219502ATB, 219503ACR, 219504ACR, 219505ACR, 219901ATB, 219904ATB, 163801ATB, 170801ATB, 231101ATB, 231102ATB |
| Behavioral disorders | Use of psychotropic medications including antipsychotics, antidepressants, and mood stabilizers | ATC code: 123102ATB, 123102ATR, 123104ATR, 123130ASY, 136401ATB, 181001ATB, 181002ATB, 181003ATB, 181004ATB, 181005ATB, 206301ATB, 206302ATB, 206303ATB, 206330ASS, 241801ACR, 241801ATB, 241802ACR, 241803ACH, 241803ACR, 241803ATB, 241804ACH, 241804ACR, 241804ATB, 247002ACS, 247701ATB, 250601ATB, 611702ATB, 611703ATB, 611704ATB, 611705ATB, 131901ATB, 131905ATB, 131908ATB, 137501ATB, 137502ATB, 137503ATB, 137504ATB, 167903ATB, 167904ATB, 167905ATB, 167906ATB, 167908ATB, 204001ATB, 204001ATD, 204002ATB, 204002ATD, 204004ATB, 204005ATB, 224201ATB, 224201ATD, 224202ATB, 224202ATD, 224203ATB, 224204ATB, 378601ATB, 378602ATB, 378603ATB, 378604ATB, 378605ATB, 378605ATR, 378606ATR, 378607ATR, 378608ATR, 378609ATR, 451501ATB, 451501ATD, 451502ATB, 451502ATD, 451503ATB, 451504ATB, 451505ATB, 451508ATB, 503201ATR, 503202ATR, 503203ATR, 161501ACH, 161501ATB, 161502ACH, 161502ATB, 161502ATD, 209301ATB, 209302ATB, 209304ATR, 209305ATR, 227001ATB, 227002ATB, 227003ATB, 474801ATB, 474802ATB, 474803ATB |
| Hormone replacement therapy (HRT) | Use of sex hormone replacement agents | ATC code: 154901ATB, 155001ATB,  155002ATB, 155401ATB, 155402ATB, 188903ATB, 188906ATB, 195001ACS |
| Diabetes insipidus | Use of desmopressin or similar agents | ATC code: 141401APD, 141401ATB, 141401ATD, 141404APD, 141404ATB, 141404ATD, 141407ATL, 141408ATL, 141409ATL, 141410ATL, 141411ATL |
| Bariatric surgery | Underwent bariatric surgical procedures | Procedure codes: Q2630–Q2639, QA630–QA647 |
| Obstructive sleep apnea (OSA) | Diagnosis with OSA and use of CPAP | ICD-10: G47.3; Device codes: MM360, MM400, MM680 |
| Adenotonsillectomy | Underwent adenoidectomy and/or tonsillectomy | Procedure codes: Q2280, Q2281, Q2290, Q2300 |
| Adrenal insufficiency | Diagnosis of adrenal insufficiency | ICD-10: E27.3, E27.4 |
